# Orientation Planning in the Fused Deposition Modeling 3D Printing of Anatomical Spine Models

**DOI:** 10.7759/cureus.7081

**Published:** 2020-02-23

**Authors:** Aaron Damon, William Clifton, Fidel Valero-Moreno, Eric Nottmeier

**Affiliations:** 1 Neurological Surgery, Mayo Clinic, Jacksonville, USA

**Keywords:** spine surgery, spine models, simulation, fused deposition modeling, facets, anatomy, 3d printing

## Abstract

Three‐dimensional (3D) printing has revolutionized medical training and patient care. Clinically it is used for patient‐specific anatomical modeling with respect to surgical procedures. 3D printing is heavily implemented for simulation to provide a useful tool for anatomical knowledge and surgical techniques. Fused deposition modeling (FDM) is a commonly utilized method of 3D printing anatomical models due to its cost-effectiveness. A potential disadvantage of FDM 3D printing complex anatomical shapes is the limitations of the modeling system in providing accurate representations of multifaceted ultrastructure, such as the facets of the lumbar spine. In order to utilize FDM 3D printing methods in an efficient manner, the pre-printing G-code assembly must be oriented according to the anatomical nature of the print. This article describes the approach that our institution's 3D printing laboratory has used to manipulate models’ printing angles in regard to the print bed and nozzle, according to anatomical properties, thus creating quality and cost-effective anatomical spine models for education and procedural simulation.

## Introduction

Three-dimensional (3D) printing is a growing tool for creating patient-specific and anatomical models for simulation and education, especially within the field of neurosurgery [1]. Fused deposition modeling (FDM) is a 3D printing process that uses a continuously extruded filament through a heated printer core. This is universally accomplished with thermoplastic materials that have varying mechanical and physical properties that can be tailored to the function of the desired print [2]. FDM 3D printing has been investigated for creating lumbar vertebral models for education and procedural simulation [3,4]. Lumbar spine pathology is the most commonly encountered diagnosis within the field of spine surgery, and the majority of existing simulations and spine models are tailored to this region of the spine [1]. During creation of these models for educational purposes, it is paramount to ensure that maximum fidelity is achieved, especially in the context of spatial relationships between adjacent anatomical structures, such as the zygapophyseal joints of the lumbar spine which are oriented in the sagittal plane, and transverse processes which are oriented in the axial plane [5]. These structures provide vital landmarks for lumbar spine surgery, both in decompression and instrumentation. In order to produce 3D printed models that have preservation of native anatomical features that allow demonstration of these relationships, maintaining precision and quality of the produced models is key to ensuring a suitable educational experience. This can be a challenge when using FDM printing methods, as the orientation of the model in relation to the build plate plays a key role in determining the quality of the print. In our experience of producing more than 1,000 anatomical models for medical education and procedural simulation, we have found that the key to producing anatomically accurate vertebral models is a comprehensive understanding of the natural anatomy of the human spine, combined with understanding of gravitational and material limitations during material extrusion for FDM 3D printing [6,7]. In this technical note, we delineate the importance of lumbar model orientation in relation to the printer bed, and provide a helpful guide for producing quality vertebral models with preservation of anatomical ultrastructure for education and simulation purposes. 

## Technical report

**Figure 1:**
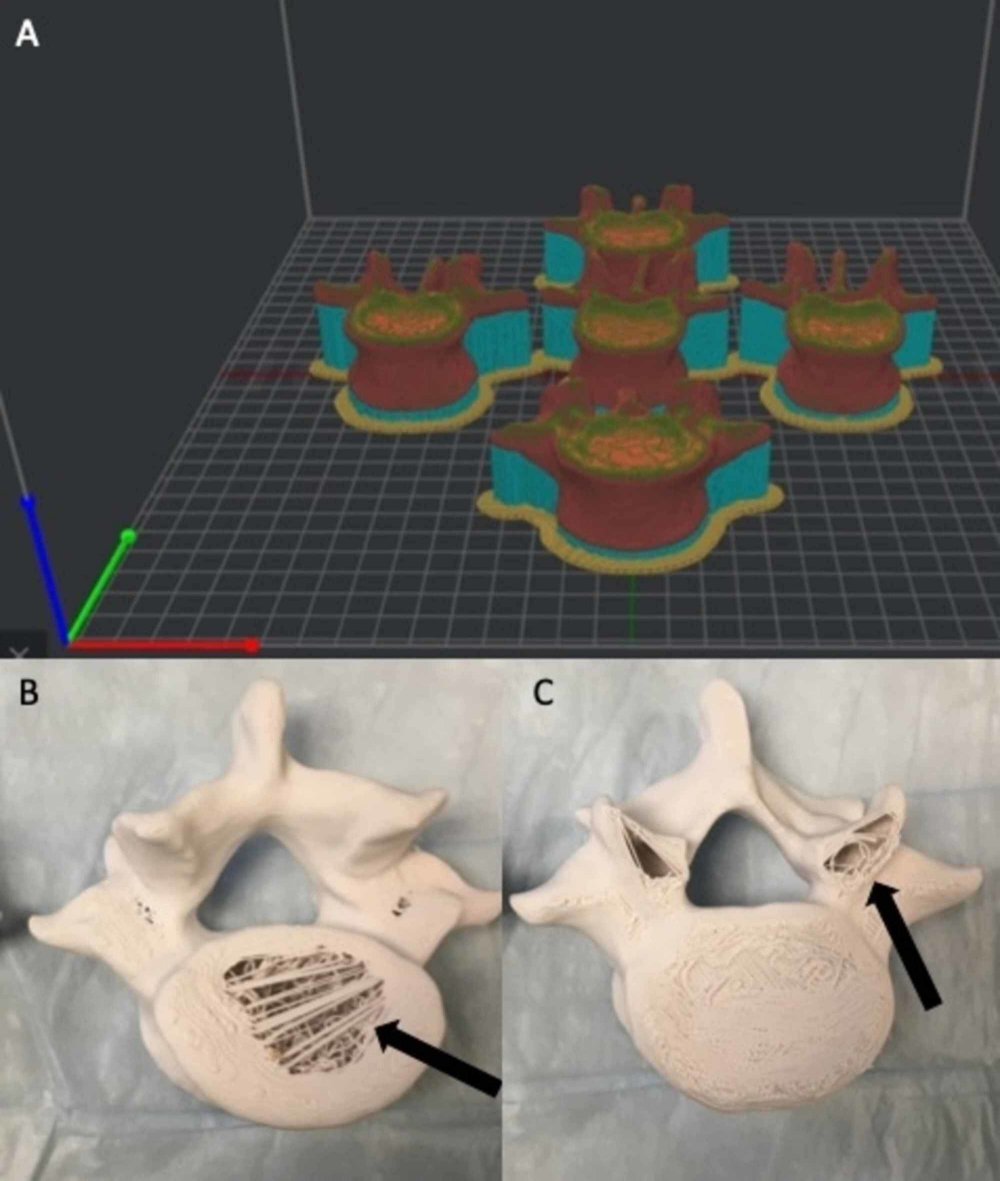
Vertebral model orientation in anatomical position in relation to the print bed. (A) The vertebral models are arranged in anatomical position with the inferior endplate parallel to the print bed within the virtual environment of the slicing software. This is the factory default setting for majority of slicing software programs. (B, C) Due to the required motion of the printer head in the X-Y plane to create the sliced model, gravitational error and material cooling caused model integrity compromise in the inferior endplate and superior facets (arrows).

With Institutional Review Board approval, an anonymized computed tomography scan of the lumbosacral spine was acquired and uploaded into an open access DICOM (Digital Information and Communications in Medicine) viewing software (3D Slicer). The anatomical structures are then highlighted and preserved using thresholding modules for Hounsfield units within the range of cortical and cancellous bone (90-3,000 units). The vertebra’s complete anatomical structures (pedicle, transverse process, spinal canal, pars interarticularis, etc.) vital to the simulator construction were inspected on each slice and preserved using software segmentation techniques. These vertebrae were inspected in a 3D projection within 3D Slicer, rendered to STL (Standard Tessellation Language) format, and then edited using Meshmixer, another open access software program. Once the individual STL files were created and edited they were uploaded into ideaMaker, a 3D slicing software. The models were rendered hollow by reducing the infill density to 0% and then arranged on the printer bed in standard anatomical position, with the inferior endplate parallel to the print bed. This was done in order to decrease the filament use for the print. The Raise3D Pro2 Plus FDM 3D printer was used for production. Twenty (20) lumbar vertebral models were printed with acrylonitrile butadiene styrene (ABS) filament using a 1 mm outer layer density to simulate cortical bone. The finished models were found to have symmetric failures on the endplates of the vertebral bodies and superior facets (see Figure 1).

**Figure 2:**
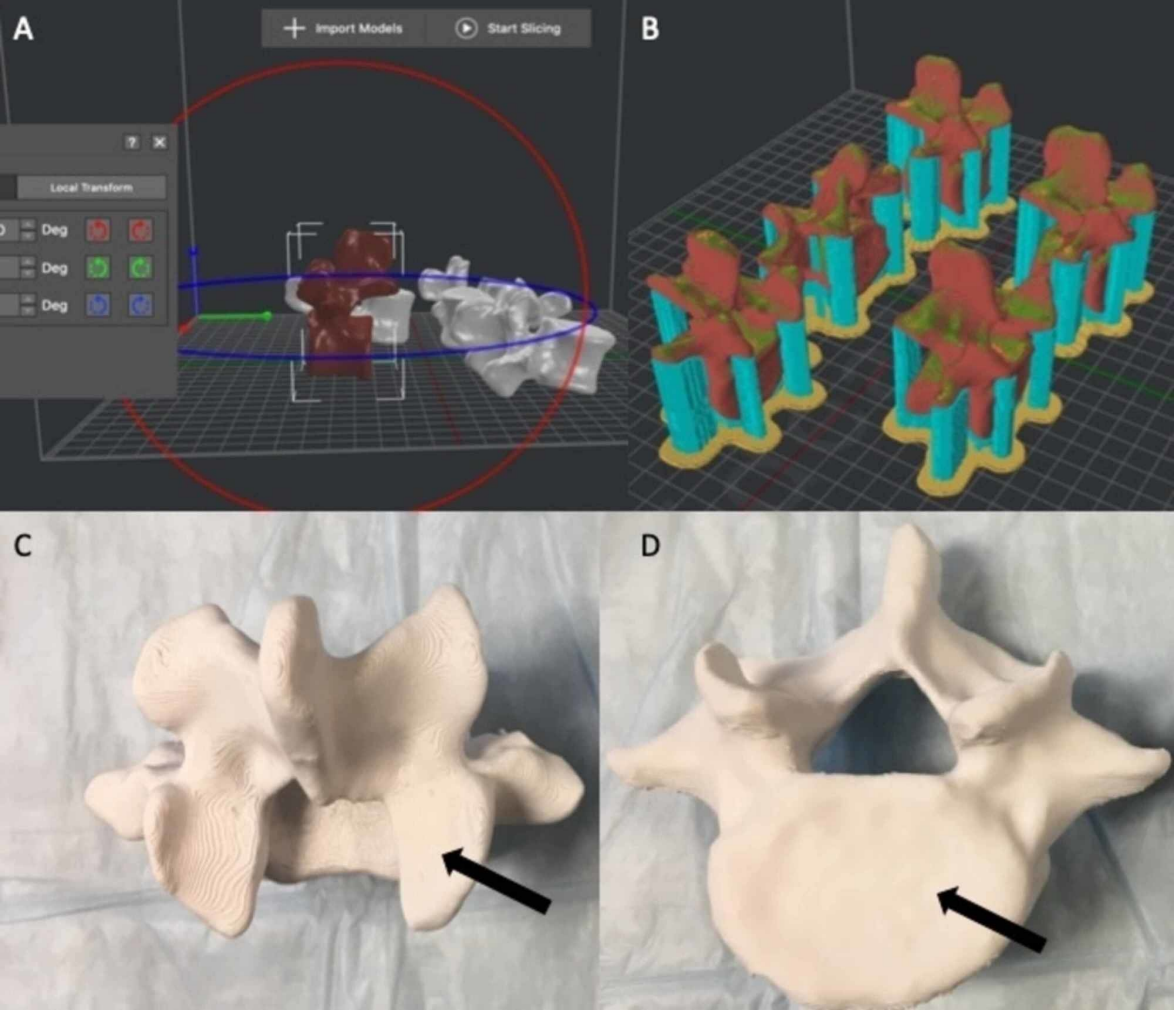
Rotation of the models to 90 degrees. (A, B) Rotating the models 90 degrees within the virtual environment before printing allowed the majority of model volume to be oriented in the Z plane, thus limiting the need for excessive motion of the printer head in the X and Y planes. (C, D) The previous print errors were corrected using this technique and the finished models demonstrated no integral errors in representative anatomy (arrows).

The individual STL files were then re-sliced and rotated 90 degrees on the simulated build plates within the 3D software, leaving less surface area in the horizontal plane (see Figure 2). This was theorized to improve the quality of the print due to the decreased need for printer head movement in the X and Y planes. Twenty (20) lumbar spine models were again printed using the same material composition and parameters. After printing the models in this orientation, the print quality defects that were previously seen in the horizontally oriented lumbar prints were resolved.

## Discussion

FDM uses a filament that is fed from a large coil through a moving printer extruder head. This heated printer head liquefies the thermoplastic and deposits it on the printer bed, which moves in the Z plane in order to provide height to the desired print. The print head is moved under a defined coding sequence to form the printed shape in the X and Y planes. The speed of the extruder may also be controlled to form an interrupted plane without stringing or dribbling between sections. The head moves in two dimensions (X-Y) to deposit one horizontal plane, or layer, at a time, and the print bed is then moved vertically (Z) by a small amount to begin a new vertical layer. Layers are stacked to create a defined shape and or model. The FDM printer is capable of creating high-end prototypes or medical models, but requires the operator to perform calibration for component speeds and temperatures to get the best results. This has led to the argument that FDM printers can provide more nuisance than benefit due to the requirement for more initial programing work [8]. However, FDM printing is cost-effective and provides the ability to manipulate STL files in a virtual manner to exploit the natural properties of the filament used and the complex shape of the print [9]. The variety and continual innovation of new thermoplastics makes for an endless possibility of material selection. There are also cost benefits associated with FDM printing. Low cost materials can be used to create and refine prototypes of models before the final prints. One of the most advantageous properties of FDM printing is the ability to manipulate infill or internal density and structures, thus saving material and allowing for variability in models. This is one property that is utilized by our lab to create corticocancellous bone [10].

FDM 3D printing is increasing in popularity for medical education and simulation. The ability to reduce the amount of unnecessary material during the process of printing, such as increased infill or support material, is advantageous, especially when printing large numbers of models. In our experiment, the lumbar vertebrae required orientation more vertical and thus needed to be adjusted at a 90-degree angle to the print bed to ensure that the facets and vertebral endplates were not distorted. These initial failures in quality were caused by the basic mechanisms of FDM printing. The print head is depositing the thermoplastic at an orientation 90 degrees to the actual build plate. This allows gravity to aid in the deposition of the melted thermoplastic being extruded. The lumbar vertebrae were originally oriented parallel with the build plate. This resulted in the majority of the vertebrae being printed in the (X-Y) axis. This horizontal orientation created more travel time for the print head, allowing cooling between layers and inadequate adhesion. It also generated gravitational failures in the structures that are nearly unavoidable, unless significant infill is placed which consumes an unnecessarily large amount of material. This critically affected the quality of the print. By using the software to change the spatial location of the vertebrae to maximize efficiency of the printer head, the majority of the print was focused on the Z axis which reduced the gravitational errors with respect to the anatomical orientation on the print, thus allowing for successful model creation. 

To date, these FDM 3D printing techniques have not been explored in great detail, especially in the context of spine modeling. Though many researchers have focused on improving the mechanical properties of the models in order to simulate properties of human bone, methods to preserve the anatomical structure of the model have been investigated to a lesser extent [10-14]. The ability to manipulate the STL file before creating the G-code in order to establish the most efficient printing sequence is not a new concept. However, extensive pre-print programming is often avoided because of the time requirements and learning curve which may require multiple failures before establishing the most efficient method of printing a certain anatomical structure. However, by simply by changing the orientation of the vertebra with respect to the printed bed, the thermoplastic layers are arranged in a way that compliments the model form as well as the gravitational constraints and printer motion in order to produce a quality product. Although not explored fully in this manuscript, it would be reasonable to assume that this methodology can be implemented in a multitude of surgical simulation models. Comprehensive anatomical knowledge is necessary when arranging or orienting models on the print bed in order to retain form. 

## Conclusions

By changing the orientation angles of the vertebrae on the print bed with respect to the nozzle and native anatomical features, our experiment maintained the critical anatomical components on hollow lumbar vertebral 3D models without unnecessary structural infill. Spatial orientation with respect to the build plate and extruder is critical to produce anatomically correct simulators used in surgical education. By implementing these simple techniques on FDM 3D printers and sharing it universally, we hope to provide a method for others to create cost-effective and anatomically accurate 3D printed lumbar spine models. 
